# Hydrotherapy in Tuberculosis

**Published:** 1907-05

**Authors:** 


### HYDROTHERAPY IN TUBERCULOSIS.

Of 1,000 tuberculosis patients treated by hydrotherapeutic measures by D. Kuthy (Blatter fur kli. Hydrother, and Therapeutical Review), in Budopest, only 15 per cent. were discharged without any improvement, while from Belzig, at Berlin, 42.5 per cent. were discharged thus; of his patients admitted in the first stage, 53.9 per cent. were discharged clinically cured; of the Belzig patients only 39 per cent. There is but one difference in the treatment as carried out in the two institutions, at Belzig douches are the only hydrotherapeutic measures employed; at Budapest each individual receives any form of hydrotherapy deemed likely to prove beneficial. The patients well enough to be about are treated most frequently with the half-bath; the patients who are kept in bed are treated by cold washing of the entire body in the morning and cold compresses to the trunk, changed every two or three hours. The duration of the half-baths three minutes, the temperature of the water at first is 89° F., and is gradually reduced to 82° F.; while the patient is in the water, he is well rubbed. The treatment improves the appetite, regulates the bowels, acts as a tonic, improves the sleep, betters the subjective state and is a mild hardening agent; with neurasthenics the water must not be below 5° F., otherwise they become irritable and restless. The bath is not used to reduce fever; patients with fever are better kept quietly in bed, nor would it be good to remove from them too large quantities of heat, as their fever is due to a breaking down of tissues; the cold water increases cough; such patients are treated to much greater advantage with the cold water compresses. The entire trunk may be covered with a cold, wet bed-sheet and this covered with a dry one and the patient thus placed in an evaporation bath. If adhered to for some time, patients even in the third stage will have their fever reduced to normal and feel themselves much better; their appetite often improves remarkably. The cold wash of one part after the other every morning takes the place of the half-bath above mentioned. Douches are used by him but little; they are altogether contra-indicated in neurasthenics, where there is a tendency to fever production, tendency to pulmonary hemorrhage and pleural pain. Foot baths and cold applications to the calves are good to reduce the temperature and induce sleep. Cold applications over the heart reduce cardiac irritability, just as warm applications over the stomach reduce gastric irritability.—Medical Fortnightly, March, 1907.

